# Tungsten-Based Contrast Agent for Photon-Counting Detector CT Angiography in Calcified Coronaries: Comparison to Iodine in a Cardiovascular Phantom

**DOI:** 10.1097/RLI.0000000000001073

**Published:** 2024-03-25

**Authors:** Thomas Sartoretti, Michael C. McDermott, Lion Stammen, Bibi Martens, Lukas J. Moser, Gregor Jost, Hubertus Pietsch, Ralf Gutjahr, Tristan Nowak, Bernhard Schmidt, Thomas G. Flohr, Joachim E. Wildberger, Hatem Alkadhi

**Affiliations:** From the Diagnostic and Interventional Radiology, University Hospital Zurich, University of Zurich, Zurich, Switzerland (T.S., L.J.M., H.A.); Department of Radiology and Nuclear Medicine, Maastricht University Medical Center, Maastricht, the Netherlands (T.S., M.C.M., L.S., B.M., T.G.F., J.E.W.); CARIM School for Cardiovascular Diseases, Maastricht University, Maastricht, the Netherlands (T.S., M.C.M., L.S., B.M., J.E.W.); Bayer AG, Berlin, Germany (M.C.M., G.J., H.P.); and Computed Tomography Division, Siemens Healthineers AG (R.G., T.N., B.S., T.G.F.), Forchheim, Germany.

**Keywords:** tungsten, iodine, photon-counting CT, dual-energy CT, virtual monoenergetic imaging

## Abstract

**Objectives:**

Calcified plaques induce blooming artifacts in coronary computed tomography angiography (CCTA) potentially leading to inaccurate stenosis evaluation. Tungsten represents a high atomic number, experimental contrast agent with different physical properties than iodine. We explored the potential of a tungsten-based contrast agent for photon-counting detector (PCD) CCTA in heavily calcified coronary vessels.

**Materials and Methods:**

A cardiovascular phantom exhibiting coronaries with calcified plaques was imaged on a first-generation dual-source PCD-CT. The coronaries with 3 different calcified plaques were filled with iodine and tungsten contrast media solutions equating to iodine and tungsten delivery rates (IDR and TDR) of 0.3, 0.5, 0.7, 1.0, 1.5, 2.0, 2.5, and 3.0 g/s, respectively. Electrocardiogram-triggered sequential acquisitions were performed in the spectral mode (QuantumPlus). Virtual monoenergetic images (VMIs) were reconstructed from 40 to 190 keV in 1 keV increments. Blooming artifacts and percentage error stenoses from calcified plaques were quantified, and attenuation characteristics of both contrast media were recorded.

**Results:**

Blooming artifacts from calcified plaques were most pronounced at 40 keV (78%) and least pronounced at 190 keV (58%). Similarly, percentage error stenoses were highest at 40 keV (48%) and lowest at 190 keV (2%), respectively. Attenuation of iodine decreased monotonically in VMIs from low to high keV, with the strongest decrease from 40 keV to 100 keV (IDR of 2.5 g/s: 1279 HU at 40 keV, 187 HU at 100 kV, and 35 HU at 190 keV). The attenuation of tungsten, on the other hand, increased monotonically as a function of VMI energy, with the strongest increase between 40 and 100 keV (TDR of 2.5 g/s: 202 HU at 40 keV, 661 HU at 100 kV, and 717 HU at 190 keV). For each keV level, the relationship between attenuation and IDR/TDR could be described by linear regressions (*R*^2^ ≥ 0.88, *P* < 0.001). Specifically, attenuation increased linearly when increasing the delivery rate irrespective of keV level or contrast medium. Iodine exhibited the highest relative increase in attenuation values at lower keV levels when increasing the IDR. Conversely, for tungsten, the greatest relative increase in attenuation values occurred at higher keV levels when increasing the TDR. When high keV imaging is desirable to reduce blooming artifacts from calcified plaques, IDR has to be increased at higher keV levels to maintain diagnostic vessel attenuation (ie, 300 HU), whereas for tungsten, TDR can be kept constant or can be even reduced at high keV energy levels.

**Conclusions:**

Tungsten's attenuation characteristics in relation to VMI energy levels are reversed to those of iodine, with tungsten exhibiting high attenuation values at high keV levels and vice versa. Thus, tungsten shows promise for high keV imaging CCTA with PCD-CT as—in distinction to iodine—both high vessel attenuation and low blooming artifacts from calcified plaques can be achieved.

Coronary computed tomography angiography (CCTA) is a noninvasive imaging technique that has become an important tool for the diagnosis and assessment of coronary artery disease.^1^ Coronary computed tomography angiography is exclusively based on iodine, as it is the only intravascular contrast medium approved for human CT imaging. Sufficient enhancement of the coronary vessel lumen is a prerequisite to achieving diagnostic images. Low kilovoltage (kV) imaging or spectral CT with low-energy virtual monoenergetic image (VMI) reconstructions have emerged as promising approaches to improve vessel contrast without increasing the iodine dose by closing in on iodine's k-edge at 33.2 keV.^2^

However, balancing low keV energy levels for diagnostic iodine vessel enhancement in the presence of high attenuating structures such as calcified plaques is still challenging. Although low keV images enhance iodine contrast, they also increase the calcium blooming effect.^3,4^ On the other hand, high keV images reduce calcium blooming at the expense of a reduced vessel attenuation.^5^ This effect can interfere with accurate assessment of coronary stenosis and plaque characterization.^2,3,6^ Thus, in clinical routine, a trade-off must be found between diagnostic vessel attenuation (usually around 300 Hounsfield units [HU]^7^) and minimized blooming artifacts.^6^

Beyond lowering the tube voltage or the use of low keV reconstructions, the contrast injection protocol can be optimized to achieve higher vessel attenuation for CCTA. For example, an increase in the iodine delivery rate (IDR) or the iodine dose can result in an improvement of the attenuation in the target area.^8–12^ However, usually, it is desired to keep the IDR and the contrast dose as low as reasonably possible.^13,14^

Recently, high atomic number (high-Z) contrast media such as tungsten-based agents have been proposed as an alternative for CT imaging.^5^ Higher-Z elements exhibit k-edges significantly higher than iodine (eg, tungsten with a k-edge of 69.5 keV). As a consequence, x-ray attenuation is higher at higher tube voltage settings or keV levels.^5^ This may allow for improved CT imaging at high-energy levels (high kV or high keV) without increased contrast material dose.^5,15,16^

Furthermore, within the CT x-ray energy range, iodine and calcium exhibit similar attenuation characteristics. This limits the accuracy of distinguishing iodine from calcium with spectral CT.^5,15,16^ Higher-Z elements with considerably different attenuation characteristics than calcium or iodine could improve the ability to distinguish contrast agents from calcium with spectral CT.^5,17^

Given these considerations, we explored the potential of a tungsten-based contrast agent for CCTA compared with iodine as the reference standard to improve coronary vessel attenuation in the presence of calcified plaques on VMI obtained with PCD-CT. To this extent, we performed an in vitro study using a cardiovascular phantom with a heart module containing different anthropomorphic highly calcified plaques in the coronaries.

## MATERIALS AND METHODS

### Phantom Study

**FIGURE 1:**
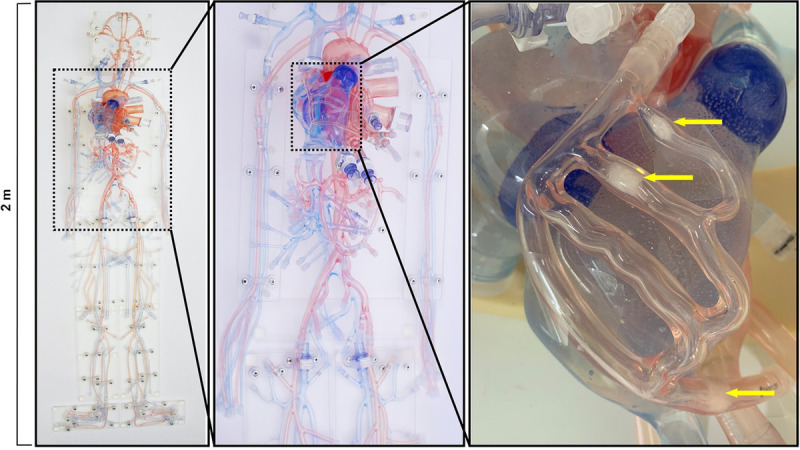
From left to right: image of full body phantom; thorax and abdomen of the phantom including the heart model; image of the coronary vessels laid on top of the heart model including yellow arrows pointing to the calcified lesions within the coronary model. Images courtesy of United Biologics Inc, Santa Ana, CA.

The cardiovascular phantom has been described previously in detail.^18^ The phantom includes interconnected cerebral, thoracic, abdominal, and peripheral vasculature with hemodynamically accurate flows; however, the focal region of this evaluation was the cardiac frame as shown in Figure 1.

The phantom includes a highly physiological heart model with 4 chambers and embedded tricuspid, pulmonary, mitral, and aortic valves. Modular connection points at the base of the aortic root lead to left and right coronary branching models overlaid on the exterior of the heart model. The coronary models are capable of being exchanged between healthy and diseased versions, including soft (ie, noncalcified) and densely calcified plaques of varying occlusion percentages. Notably, for the current study, the coronary model included 3 plaques of heterogeneous character composed of highly attenuating, deposited calcium carbonate with occlusion percentages of 50%, 60%, and 60%, respectively. In addition, an electromechanical pump (SuperPump; ViVitro Labs, Victoria, British Columbia, Canada) was connected to the phantom allowing for control over heart rate, stroke volume, and blood pressure, which together facilitate cardiac motion of the model. For image acquisition, the phantom was positioned inside an attenuating ring simulating thoracic tissue attenuation. The dynamic flow in the phantom was achieved by circulating room temperature water at a heart rate of 60 beats per minute and a stroke volume of 80 mL. Internal pressure of the phantom (ie, simulated blood pressure caused by circulating water) was maintained at 120/80 mm Hg.

### Contrast Media

We investigated the potential of the following contrast media at equal mass concentrations: iodine (iopromide, Ultravist 300; Bayer Healthcare AG, Berlin, Germany) and tungsten (W_3_O_2_ complex, manufactured for experimental purposes by Bayer Healthcare AG, Berlin, Germany).^19^ All test solutions were prepared at equal mass concentrations ranging from 2.8 to 28.4 mg of contrast medium per milliliter to simulate different contrast media delivery rates (0.3, 0.5, 0.7, 1, 1.5, 2, 2.5, and 3 g of contrast medium per second). More information on the contrast media and their spectral properties can be found elsewhere.^16,20^

In terms of contrast media, injections were first performed with a power injection (MEDRAD Centargo; Bayer AG, Berlin, Germany) at the aforementioned delivery rates (IDR/TDR) for both contrast media. This range of delivery rates was selected to cover the full expected clinical range of injected rates as well as the lower and upper extremes. Injection volume was adjusted to maintain a constant injection duration of 10 seconds. A Coriolis flow meter (MicroMotion 5700; Emerson Electric Co, St. Louis, MO) was used to measure the concentration of each contrast medium entering the coronary vessels throughout the injection duration and the following 60 seconds. The peak concentration was recorded and averaged across 5 repeated trials for each flow rate. Subsequently, the coronaries were manually filled with iodinated contrast medium or the experimental tungsten compound titrated with water to a concentration matching those calculated for the given delivery rate using the Coriolis meter. Although connected to the phantom, the coronaries were isolated from the dynamic flow through the rest of the vasculature to ensure precise concentration of contrast media in the target vessels throughout the various trials and to protect the geometry of the coronary plaques.

### CT Image Acquisition

All scans were acquired on a first-generation, whole-body, dual-source PCD-CT system (NAEOTOM Alpha, software version VA50; Siemens Healthineers AG, Forchheim, Germany) equipped with 2 photon-counting detectors (cadmium telluride). Electrocardiogram-triggered sequential acquisitions were performed in the spectral mode (QuantumPlus). Tube voltage was set at 140 kV to enable maximum spectral separation. A collimation of 144 × 0.4 mm was used, and the image quality (IQ) level was set to 60. Gantry rotation time was 0.25 seconds, resulting in a temporal resolution of 66 milliseconds. For all scans, radiation dose values were as follows: CTDI_vol_ of 3.03 ± 0.67 mGy and DLP of 53.3 ± 8.34 mGy·cm.

### CT Image Reconstruction

Virtual monoenergetic images were reconstructed from 40–190 keV in 1 keV increments both for iodine and tungsten. To calculate VMIs, the spectral data measured in the different energy bins of the PCD are combined with keV-dependent weighting factors in such a way that the correct attenuation of iodine is obtained for each keV value. These weighting factors are precomputed and stored in the CT scanner and were used for both iodine and tungsten.

Images were reconstructed with a slice thickness of 0.4 mm and an increment of 0.3 mm using quantum iterative reconstruction at strength 4 and a medium sharp vascular kernel (Bv56, Siemens). A matrix size of 512 × 512 was used, and a field of view of 200 × 200 mm^2^ was selected.

### Data Analysis

All data analyses were performed by a reader with 4 years of experience in cardiovascular imaging research. First, to quantify blooming effects of calcified plaques, linear measurements of the maximal external plaque diameter and internal lumen diameter on long axis views using manual double-oblique reconstructions were performed for all 3 plaques. Measurements were performed on the scan with the lowest TDR (ie, very low attenuation across all keV energies, similar to water), and the window level was fixed at 600/150 for all keV levels (40 to 190 keV in 10 keV increments) corresponding to the default setting of the software program (Syngo.via, version VB80A; Siemens Healthineers AG, Forchheim, Germany). Blooming artifacts were subsequently approximated as previously defined using the measurements and the following formula:^21^


$$\text{Blooming artifact}\left(\%\right)=\begin{array}{l} \frac{\text{External calcified plaque diameter}-\text{Measured lumen diameter}}{\text{External calcified plaque diameter}}\\ \times 100 \end{array}$$

In addition, using the same measurements and the vendor's specifications regarding plaque geometry, the discrepancies between measured stenoses and true stenoses were computed (named percentage error stenoses) as follows:


$$\text{Error stenosis}\;\left(\%\right)=\left(1-\left(\frac{\text{Measured lumen diameter}}{\text{True lumen diameter}}\right)\right)\times 100$$

For subsequent statistical analysis, the data of all 3 plaques were averaged.

Second, the reader placed circular regions of interest into the proximal part of the largest coronary vessel of the coronary model for each image series. The mean attenuation was recorded and was used as a marker for vascular enhancement.

### Statistical Analysis

All analyses were performed using R statistical software (R Core Team, version 4.1.1; R Foundation for Statistical Computing, Vienna, Austria). The package “ggplot2” was used to visualize the data, whereby the data were fitted to the various endpoints (ie, attenuation, etc) stratified by the target variables (ie, keV energy, IDR/TDR, etc). Linear regression models were computed and fitted to the data. *R*^2^ values were considered as markers for goodness of fit. Data are reported as mean or median depending on the data distribution. Two-sided *P* values <0.05 were considered statistically significant.

## RESULTS

### Blooming Artifacts and Percentage Error Stenoses

**FIGURE 2:**
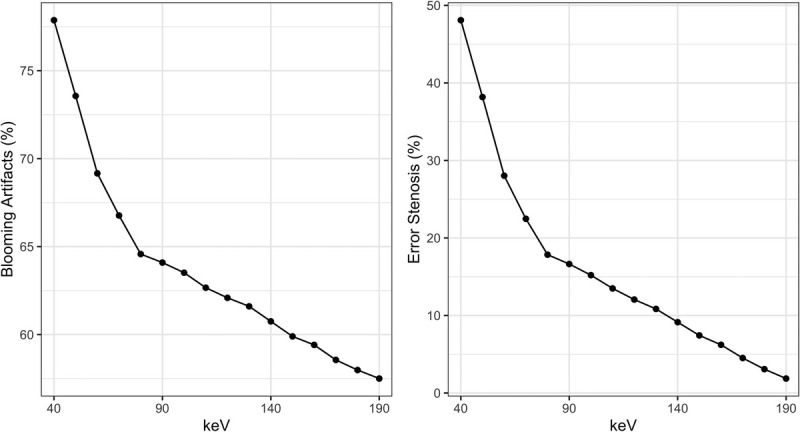
Overview of blooming artifacts (A) and percentage error stenoses (B) stratified by keV energy level.

Blooming artifacts were most pronounced at 40 keV and least pronounced at 190 keV, respectively. Specifically, blooming dropped from 77.9% at 40 keV to 63.5% at 100 keV and to 57.5% at 190 keV. A similar trend was found for percentage error stenoses. Specifically, percentage error stenoses dropped from 48.1% at 40 keV to 15.2% at 100 keV to 1.9% at 190 keV. A detailed overview is provided in Figure 2.

### Attenuation Characteristics

Irrespective of the delivery rate, attenuation of iodine decreased monotonically in VMIs from low to high keV, with the strongest decrease from 40 keV to 100 keV (exemplarily at an IDR of 2.5 g/s: 1279 HU at 40 keV, 187 HU at 100 kV, and 35 HU at 190 keV). The attenuation of tungsten, on the other hand, increased monotonically as a function of VMI energy, with the strongest increase between 40 and 100 keV (exemplarily at a TDR of 2.5 g/s: 202 HU at 40 keV, 661 HU at 100 kV, and 717 HU at 190 keV).

**FIGURE 3:**
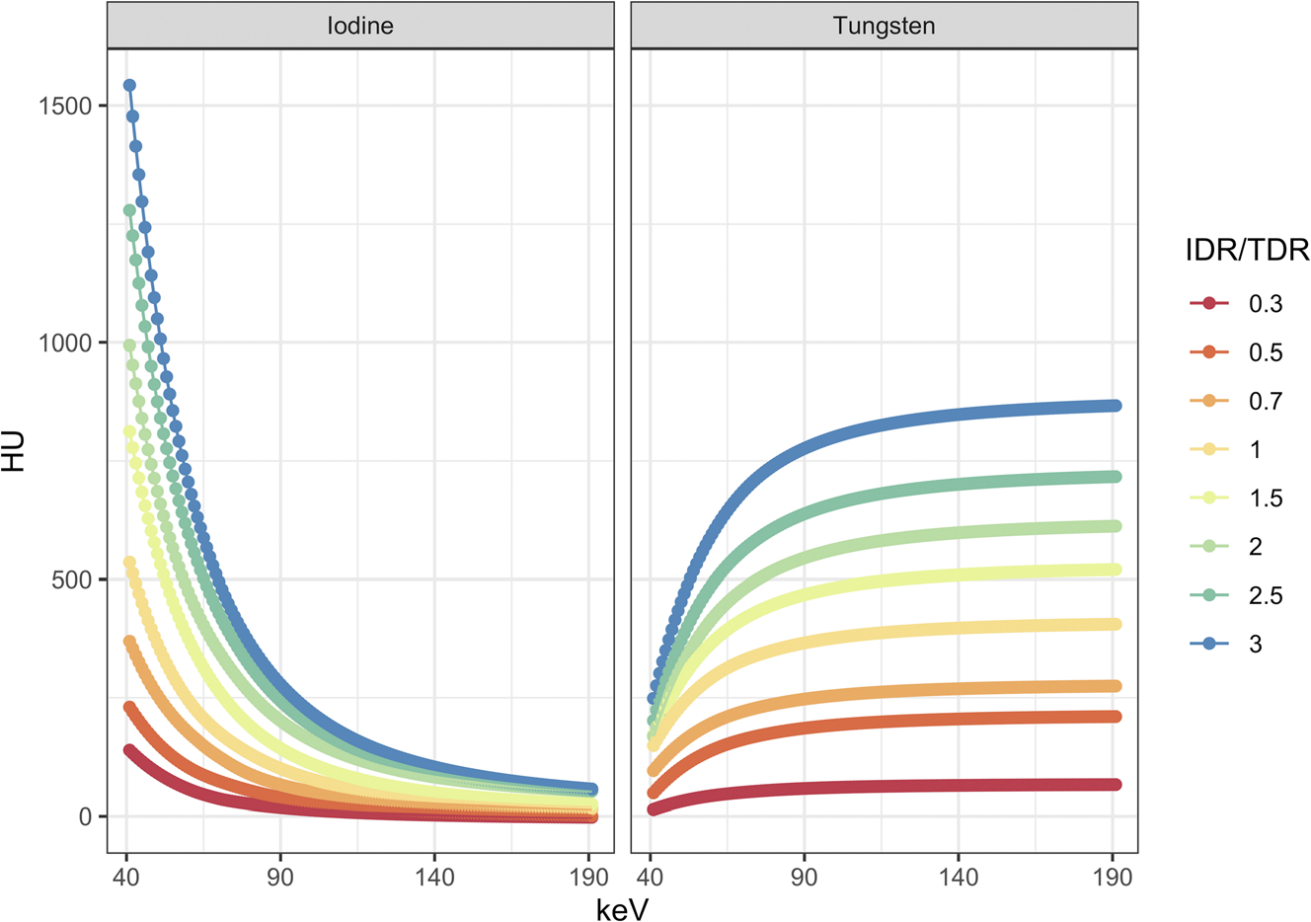
Overview of attenuation characteristics of iodine and tungsten at various delivery rates.

**FIGURE 4:**
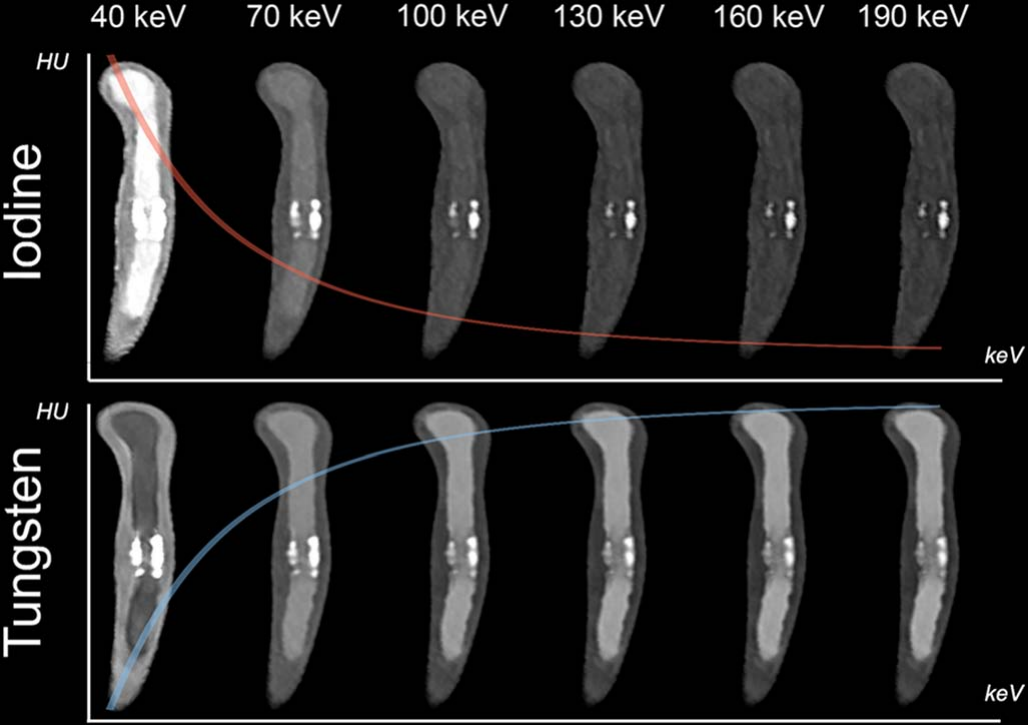
Overview of attenuation characteristics of iodine and tungsten at a delivery rate of 2.5 g/s for a coronary vessel with a calcified plaque. VMIs are (40 to 190 keV) shown in 30 keV increments. Both contrast media and all keV levels are displayed with window settings centered at 500 HU and width 1700 HU. Note the increased attenuation of iodine at lower keV levels and the increased attenuation of tungsten at higher keV levels as shown schematically by means of overlaid attenuation curves. In addition, decreasing blooming artifacts can be seen when moving from lower to higher keV levels.

A detailed overview of the attenuation characteristics of both contrast media at the various delivery rates is provided in Figure 3. In addition, an example of vascular enhancement is provided in Figure 4.

### Relationship Among Attenuation, VMI Energy Level, and Contrast Medium Delivery Rate

**FIGURE 5:**
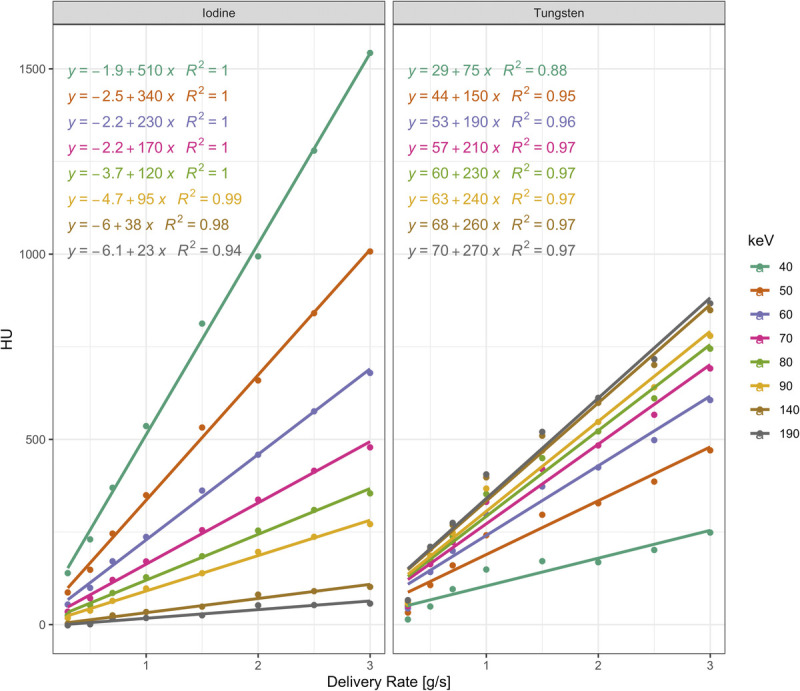
Overview of the relationship among attenuation, VMI energy level, and contrast medium delivery rate (IDR/TDR). Exemplarily, a range of VMI energy levels spanning from 40 to 190 keV are shown. Linear regression equations for each keV level and contrast medium allow for the computation of the required delivery rate to achieve a specific attenuation.

Based on our experiments and measurements, the relationship among attenuation, VMI energy level, and contrast medium delivery rate can be modeled. Notably, for each keV level, the relationship between attenuation and delivery rate can be described best by means of linear regression (*R*^2^ ≥ 0.88, *P* < 0.001) with attenuation increasing linearly when increasing the delivery rate irrespective of keV level or contrast medium. For iodine, attenuation values relatively increase the most at lower keV levels when increasing the IDR. For tungsten, attenuation values relatively increase the most at higher keV levels when increasing the TDR. A graphical overview of these relationships including the linear regression equations is provided in Figure 5.

**FIGURE 6:**
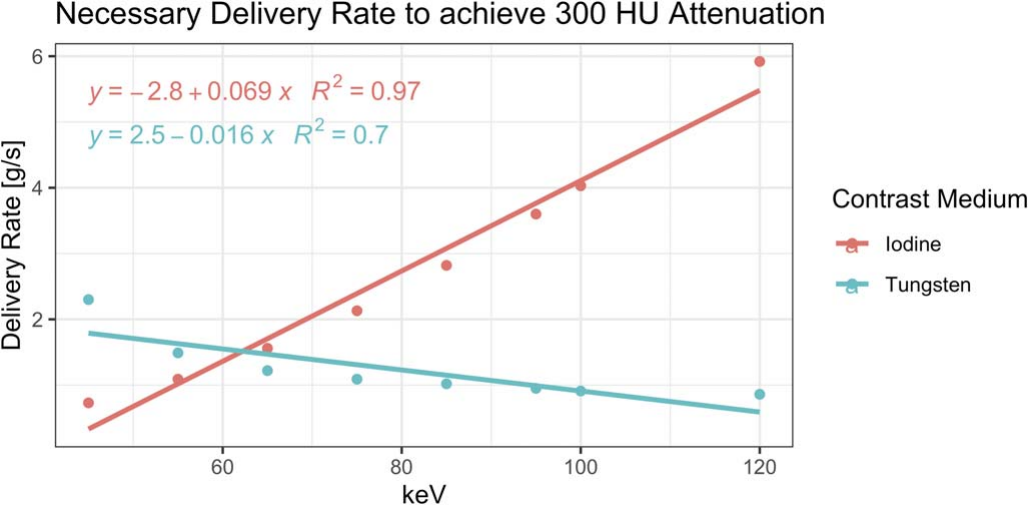
Relationship between VMI energy level and delivery rate for iodine and tungsten to achieve an attenuation of 300 HU in the vessel lumen.

This relationship can be used to compute the optimal delivery rate to achieve a diagnostic vessel attenuation (exemplarily 300 HU) for each contrast medium and keV level (Fig. 6). Specifically, for iodine, the use of high keV imaging requires the user to considerably increase the IDR to still achieve diagnostic attenuation, whereas for tungsten, the TDR can be kept relatively constant (or can even be decreased) across the keV levels thereby still ensuring diagnostic attenuation values.

## DISCUSSION

In this study, we assessed the potential of an experimental tungsten-based contrast agent for coronary PCD-CT angiography. Our data indicate that tungsten exhibits several favorable properties as compared with iodine that allow it to achieve both adequate diagnostic vessel attenuation and lower calcium blooming in calcified coronary vessels.

We used a first-generation dual-source PCD-CT system. This system has been shown to significantly outperform third-generation dual-source energy-integrating detector CT systems in terms of spatial resolution, noise characteristics, and dose efficiency.^22–25^ Moreover, the detector inherently enables spectral CT imaging, thus enabling the user to reconstruct spectral images from every scan, without compromising temporal resolution. Consequently, VMIs at certain energy levels have replaced polychromatic images as the standard image for image interpretation.^22,25^ This highlights the need to specifically assess the value of novel contrast agents such as tungsten for VMI.

The most important difference between iodine and tungsten is their attenuation characteristics. Although iodine exhibits sharply declining attenuation when moving from lower to higher keV levels, tungsten presents, relatively speaking, with a more constant attenuation curve across a wider range of keV levels, whereby higher attenuation values can be found at higher keV levels. Thus, with tungsten, diagnostic vessel attenuation can be achieved across a considerably wider variety of keV levels, whereby higher keV levels are favored.

Therein, it should be noted that the keV-dependent behavior of the x-ray attenuation of tungsten is a consequence of the use of keV-dependent weighting factors optimized for iodine when generating the VMIs.

In contrast to iodine, tungsten has a lower x-ray attenuation in the low-energy bin data of the PCD-CT than in the high-energy bin data. This explains why the keV-dependent shape of the attenuation curve is reversed compared with iodine, and why the absorption of tungsten in the VMIs increases with higher keV energies as observed in this work. Besides, the use of iodine weighting factors for tungsten also explains the absence of the tungsten K-edge at 69.5 keV in the VMIs.

Based on these attenuation characteristics, tungsten can potentially outperform iodine for the accurate visualization of coronaries presenting with highly calcified plaques. Specifically, by using higher keV energies, blooming artifacts from plaques can be reduced (as shown in this study), thus potentially improving stenosis quantification accuracy while simultaneously improving vessel visualization due to the increasing attenuation values at higher keV energies. Iodine, on the other hand, requires a balancing act between sufficient coronary attenuation on the one hand and minimizing artifacts from calcified plaques on the other hand, for example, using low keV energies. Furthermore, at higher keV, image noise in VMIs is reduced compared with lower keV, which additionally improves the tungsten contrast-to-noise ratio and thus also image quality.^2,22,26,27^ This is crucial in low radiation and low contrast situations as encountered in clinical practice. This again emphasizes the advantages of high keV imaging and thus also of tungsten, which enables high-quality imaging in the high keV range.

An important aspect also concerns the contrast medium injection protocol. In patients with severely calcified plaques where high keV imaging would be favorable to reduce blooming, an increase in the IDR could be a viable option to counteract the reduced attenuation of iodine at these keV energies. Our data, however, show that an increase of the IDR is particularly beneficial at lower keV energies and that the benefit then rapidly drops off at higher keV energies. In other words, when using higher keV energies, IDR has to be increased over proportionately in comparison to lower keV energies to achieve the same increase in attenuation. Therefore, optimizing the injection protocol for iodine can be valuable when using a new keV-dependent adaptation, but it remains less effective at higher keV energies where improvements are most crucial. As an example, for coronaries with severe calcium burden, a keV energy of 100 keV and upwards may be helpful to reduce blooming and to improve stenoses grading accuracy.^28,29^ This is further corroborated by the fact that, as compared with 40 keV, percentage error stenoses dropped from 48% to 15% at 100 keV in our study. However, to achieve a diagnostic attenuation of 300 HU at 100 keV, an IDR of 4 g I/s is required, which in turn would equate to a flow rate of 13.3 mL/s when using a contrast medium with a concentration of 300 mg/mL. This is clinically speaking very challenging. Optimization is therefore demanding and to a certain degree even limited.

In contrast, tungsten's attenuation behavior is reversed to that of iodine. An increase of the delivery rate of tungsten increases the attenuation to a lesser extent than iodine in absolute terms (irrespective of the keV energy). Furthermore, across all keV energies, the benefit is still highest at higher keV energies. Thus with tungsten, high keV energies are favored even further as increased delivery rates lead to over proportionately high gains in attenuation.

In other words, the adaptation of the delivery rate is more useful for iodine because the delivery rate has a larger influence on the attenuation of iodine than that of tungsten in absolute terms. Importantly, however, for tungsten, a fixed delivery rate across all keV levels seems feasible, without compromising diagnostic vessel attenuation in the coronaries.

In this investigation, we specifically focused on tungsten's benefits for VMI in the context of CCTA. However, with its radically different attenuation characteristics than calcium, tungsten may also improve dual-energy applications, such as computationally removing plaques from vessels or to assess plaque composition by means of spectral separation.^15,16^ These are interesting and clinically relevant applications that should be investigated in the future. Furthermore, the potential of tungsten for the imaging of patients with coronary stents should be emphasized. Stents pose similar challenges as calcified plaques, because they have similar attenuation characteristics and may thus also cause artifacts obscuring the vessel and in-stent lumen, especially at lower keV levels.^30^ These are relevant challenges that have until now prohibited CCTA to function as a go-to imaging modality for patients presenting with problems related to stents such as in-stent restenosis.^30,31^

Lastly, it should be noted that there are further high atomic number contrast agents that may be of value for CT imaging. Apart from tungsten, the lanthanides (ie, gadolinium and holmium among others), tantalum, hafnium, or bismuth could play an important role. Select experimental studies have shown the potential of some of these contrast agents for improved contrast-enhanced CT imaging relative to iodine, for example, in terms of providing improved calcium subtraction and stenosis assessment in small vessels^16^ or in terms of radiation dose reduction.^32^ Future studies investigating the role of these other contrast agents are warranted and of great interest.^5,15,16^ For the future, it also has to be determined whether these compounds are nontoxic and can thus be used in an in vivo setting. Some heavy metals such as iron (Fe), manganese (Mn), or tungsten (W) are considered nontoxic. The critical aspect for parenterally administered compounds lies in the molecular form of the elements and the overall tolerance of these molecules. For instance, gadolinium, although toxic as an ion, is well-tolerated in its chelated form, making it suitable as a magnetic resonance imaging contrast agent. The essential factor is ensuring the molecule's tolerability, particularly its noninteraction with biological membranes, physiological processes, and inability to enter internal cells. For tungsten-based compounds as proposed here, it can be confirmed that these important prerequisites are given.

The current investigation had several limitations. First, this was an experimental, in vitro investigation. It remains to be determined whether our findings can be translated to an in vivo clinical setting. In this regard, it should be emphasized that tungsten is not yet clinically available for use in humans. Second, we only used one dedicated scan mode and a limited set of reconstruction settings. These settings may have impacted our measurements and thus our conclusions. Importantly, we focused specifically on the use of VMI as these are currently the standard images on PCD-CT. However, we are aware that there is a further very promising scan mode for CCTA, that is, ultra-high-resolution imaging, which, however, uses conventional polychromatic images.^33–35^ In addition, we are aware that there are more advanced imaging methods such as virtual removal of calcified plaques. We did not evaluate these methods because they are currently optimized for iodine and cannot be used for contrast agents with significantly different spectral behavior—such as tungsten—without changes to the algorithm. We acknowledge that this limits the generalizability of our results, and future studies have to determine the value of tungsten for CCTA when using other scan modes or imaging methods. Finally, our coronary model only exhibited a limited set of calcified plaques with intermediate stenoses. In the future, an investigation of the value of tungsten for the visualization of soft plaques and for other calcified and mixed plaques of varying stenosis grades should be pursued.

In conclusion, this study investigated the potential of an experimental tungsten-based contrast agent for CCTA. Our data indicate that tungsten may overcome some key limitations of iodine in terms of providing diagnostically valuable images for CCTA across a variety of complex scenarios including the presence of heavily calcified plaques. The insights gained from this study should further motivate the development of tungsten as a novel CT contrast agent to be carried forward into clinical usage.
